# NMD Classifier: A reliable and systematic classification tool for nonsense-mediated decay events

**DOI:** 10.1371/journal.pone.0174798

**Published:** 2017-04-03

**Authors:** Min-Kung Hsu, Hsuan-Yu Lin, Feng-Chi Chen

**Affiliations:** 1 Department of Biological Science and Technology, National Chiao-Tung University, Hsinchu City, Taiwan; 2 Institute of Population Health Sciences, National Health Research Institutes, Zhunan Township, Miaoli County, Taiwan; 3 School of Dentistry, China Medical University, Taichung City, Taiwan; Michigan State University, UNITED STATES

## Abstract

Nonsense-mediated decay (NMD) degrades mRNAs that include premature termination codons to avoid the translation and accumulation of truncated proteins. This mechanism has been found to participate in gene regulation and a wide spectrum of biological processes. However, the evolutionary and regulatory origins of NMD-targeted transcripts (NMDTs) have been less studied, partly because of the complexity in analyzing NMD events. Here we report NMD Classifier, a tool for systematic classification of NMD events for either annotated or *de novo* assembled transcripts. This tool is based on the assumption of minimal evolution/regulation–an event that leads to the least change is the most likely to occur. Our simulation results indicate that NMD Classifier can correctly identify an average of 99.3% of the NMD-causing transcript structural changes, particularly exon inclusions/exclusions and exon boundary alterations. Researchers can apply NMD Classifier to evolutionary and regulatory studies by comparing NMD events of different biological conditions or in different organisms.

## Introduction

Nonsense-mediated decay (NMD) is a molecular mechanism whereby potentially defective messenger RNAs (mRNAs) are degraded. The term “nonsense” refers to the type of mutation (*i*.*e*. nonsense mutation, or a mutation that results in generation of a stop codon) that induces this mechanism. According to the “scanning model” of protein translation, the translation machinery scans an mRNA from the translation start site downwards until it encounters a stop codon and decouples from the mRNA [1–4]. However, if the translation machinery detects a premature translation-termination codon (PTC), it starts to recruit the NMD machinery, which then serves to degrade the “problematic” mRNA to avoid yielding a truncated peptide [1].

NMD has been conventionally regarded as an important mechanism for mRNA quality control. NMD-targeted transcripts (NMDTs) could result from point mutations, insertions/deletions, or alternative splicing events that give rise to a PTC [5,6]. NMD is observed in all investigated organisms, from bacteria to mammals [1,7,8]. This mechanism is involved in gene regulation and a wide spectrum of biological processes [9–11]. Importantly, NMD has been associated with human diseases [12–17]. For instance, Ullrich disease, an autosomal recessive congenital muscular dystrophy, has been found to be regulated by NMD factors [18,19]. Despite the biomedical importance of NMD, the evolutionary and regulatory origins of NMDTs have been less explored [20,21].

One major regulatory source of NMDT is alterations in transcript structure [22–25]. This is because inclusion/exclusion of coding exons or changes in exon boundaries may result in frameshift events, which in turn can generate PTCs. Of note, transcript structural alterations *per se* may be influenced by other mechanisms such as splice site mutations or structural variations in the genome. As mentioned above, NMD is mainly a translation-dependent mechanism. When the translation machinery halts at the first stop codon, and the stop codon is located more than 50–55 nucleotides (NTs) upstream of the last exon-exon junction, the NMD machinery will be engaged to initiate degradation of the mRNA [23,26]. This stop codon is defined as a PTC. Notably, however, exceptions to this rule have been reported. A transcript may be degraded even when the PTC is located within 50 NTs from the last exon junction (*e*.*g*. T cell receptor β-transcript), or be resistant to degradation when the PTC is far upstream (e.g. PTCs within β-globin exon 1) [2].

Evolutionary and regulatory studies of NMD require correct classification of NMD events. For example, it has been reported that the conservation level of many exon-inclusion-caused, but not exon-exclusion-caused NMD events have emerged and been conserved in placental mammals [25]. Meanwhile, intron-retention-caused NMD events have been reported to regulate gene expression in retinitis pigmentosa and Taybi-Linder syndrome [27]. Despite the importance of NMD classification, there have been no publicly available tools to serve this purpose.

Here we report NMD Classifier, a tool for systematic classification of NMD events. NMDTs have been suggested to emerge during the evolution of vertebrates because of changes in splicing patterns [25] or point mutations [5,28]. NMDTs are also observed to result from single nucleotide polymorphisms in the human population [29]. Theoretically, an evolutionary/regulatory event that involves the smallest number of changes is the most likely to occur. We thus develop the NMD Classifier on the assumption of “minimal evolution/regulation”. We hypothesize that an NMDT has resulted from an evolutionary or regulatory event that alters the reading frame of a non-NMDT (*i*.*e*. a “normal” coding transcript). By comparing an NMDT against its most similar coding transcript isoform, we could identify the transcript structure-altering event that has led to the NMD event. Our simulation results indicate that NMD Classifier yields highly accurate results in the identification of NMD-causing changes in transcript structure. This tool will be useful for future NMD-related studies, and is available at https://sourceforge.net/projects/transcriptome-analysis/files/NMD_Classifier.tar.gz

## Result

### Overview of NMD classifier

**Fig 1:**
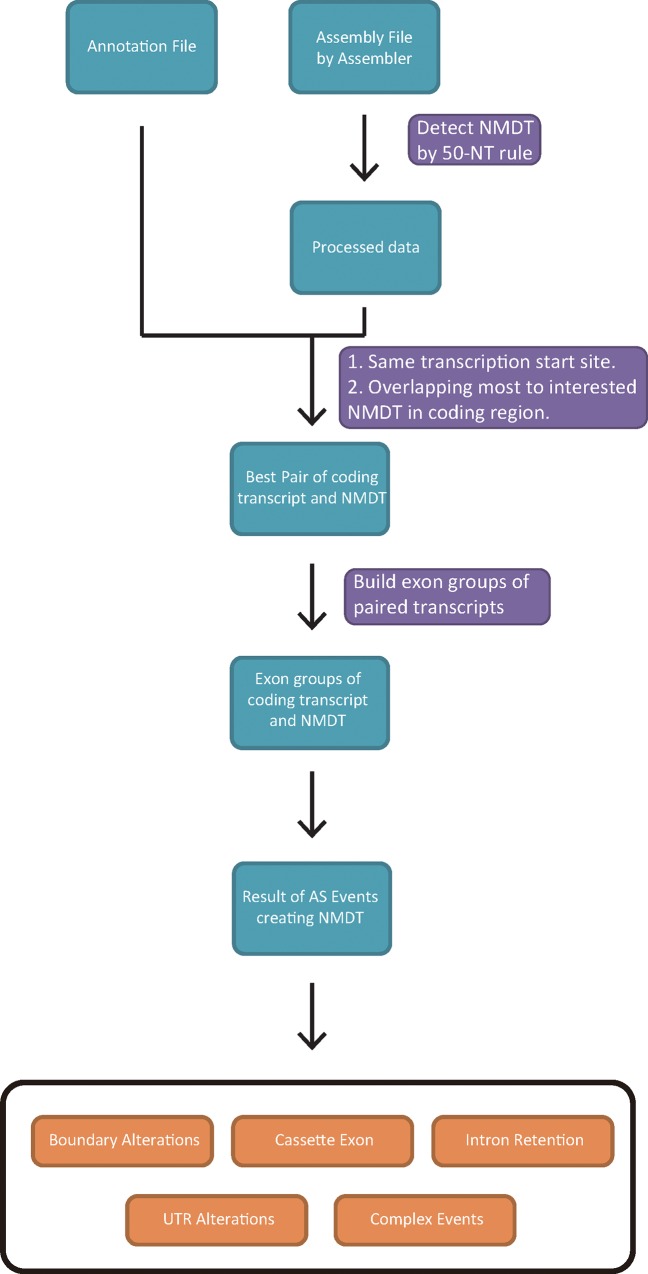
The analysis flow of NMD Classifier.

The analysis flow of NMD Classifier is shown in Fig 1. The analysis starts with input data, which are either user-generated transcript assembly annotations (in GTF format) or annotation files from Ensembl (in GTF format) or NCBI (in GFF format). For user-generated transcript assembly, NMD Classifier detects NMDTs according to the 50-NT rule (see the next section) before analyzing transcript structural changes. For Ensembl/NCBI annotation files, the NMDT detection step is optional. NMD Classifier by default skips the detection step, and takes the annotated NMDTs for classifications. Next, for each NMDT, NMD Classifier identifies the best matching coding transcript isoform (“best partner”, see Methods), which supposedly is the splicing isoform most similar to the interested NMDT. Each NMDT is then compared against its best partner. Each exon from an NMDT is “grouped” with an exon (or exons) from its best partner if the corresponding genomic regions of these exons overlapped with each other by at least one nucleotide. NMD Classifier then scans for frameshift events starting from the first exon group (the one that contains the translation start codon). If an upstream frameshift event is “rescued” by a downstream event (or events), the search for frameshift re-initiates downstream of the rescue event and continues until the last PTC is detected. Except in complex NMD events, the first transcript structure-altering event that results in the non-rescued frameshift event is considered as the cause of NMD, and is classified according to the splicing type of the specific event. In cases where no NMD-causing events are identified between an NMDT and its best partner, the NMD event is classified as “UTR alteration”. If an NMD event is caused by multiple types of transcript structural changes, it is classified as a “complex event” (Methods).

### Detection of NMDTs

**Fig 2:**
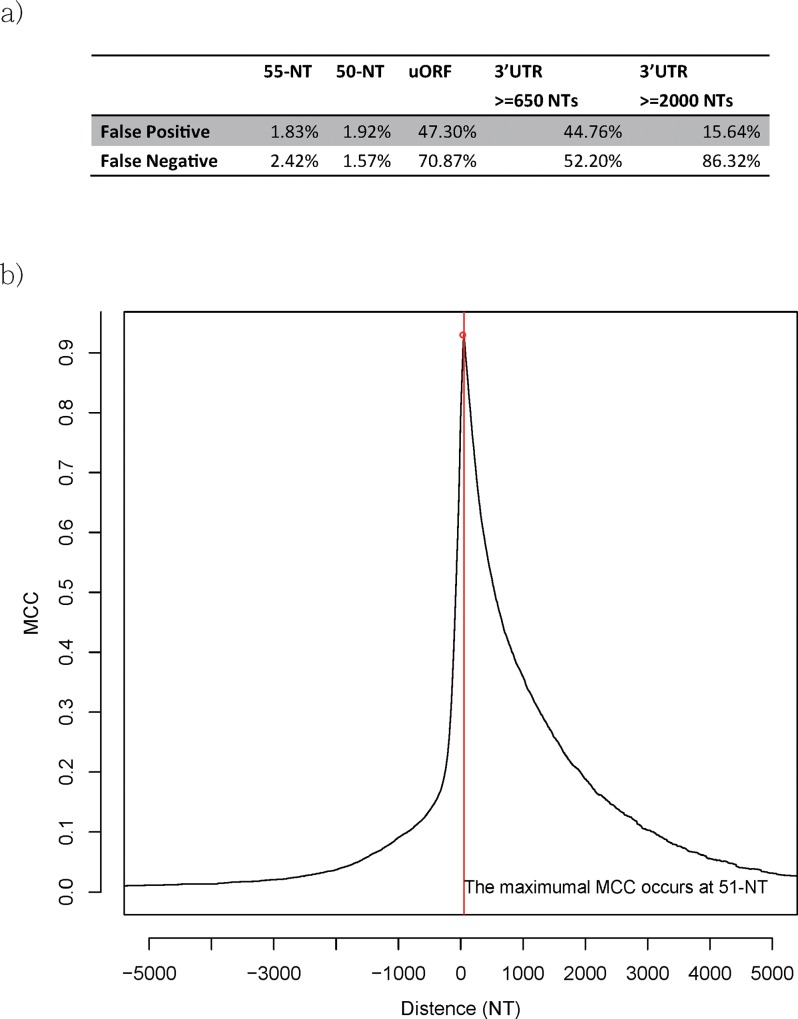
(a) The distribution of MCC values across different distances between a PTC and the last exon-exon junction. A positive (negative) distance indicates that the PTC is located upstream (downstream) of the last exon-exon junction; (b) False positive and false negative rates of different NMD prediction rule.

Ensembl and NCBI annotation files include NMDT annotations. However, if the user performs *de novo* transcriptome assembly, we must first correctly detect NMDTs before we can classify them. To this end, we have tested several commonly recognized NMDT detection criteria and used the Ensembl NMDT annotations (version 75) as a gold standard. Ensembl-annotated NMDTs are mostly supported by experimental evidence. The strength of supporting evidence is shown as the “Transcript Support Level (TSL)”. Approximately 56% of the Ensembl-annotated NMDTs have at least one supporting EST (TSL 1–3; S1 Table). The tested NMD-detection criteria include a PTC located 50 NTs or 55 NTs upstream of the last exon junction, the presence of an upstream open reading frame (uORF), and inclusion of a long (> 650 NTs or > 2000 NTs) 3’untranslated region (3’UTR) (Fig 2A) [30]. We applied these NMDT detection criteria to all of the transcripts of coding genes annotated in Ensembl V75 (including 21,037 coding genes that encoded 42,637 coding transcripts and 9,357 NMDTs). An annotated coding transcript erroneously detected as an NMDT was defined as a false positive, while an annotated NMDT not detected was considered as a false negative. Fig 2A also shows that the 55-NT rule yielded a 1.83% false positive rate and a 2.42% false negative rate. The corresponding rates of the 50-NT rule were 1.92% and 1.57%, respectively. Both of the 55-NT and 50-NT rule yielded an overall accuracy of 97.8%. In comparison, detection based on the presence of uORFs or 3’UTR length yielded unacceptably high false positive and negative rates.

To more precisely determine which PTC-last exon junction distance was the best for detecting NMDTs, we calculated the Mathews Coefficient of Correlation (MCC)[31] for different distances. Interestingly, the largest MCC value occurred at 51 NT (Fig 2B), which was very close to 50 NT. According to these results, we selected the 50-NT rule to detect NMDTs for *de novo* assembled transcripts, and integrated it into NMD Classifier.

### Evaluation of the accuracy of NMD classifier

**Fig 3:**
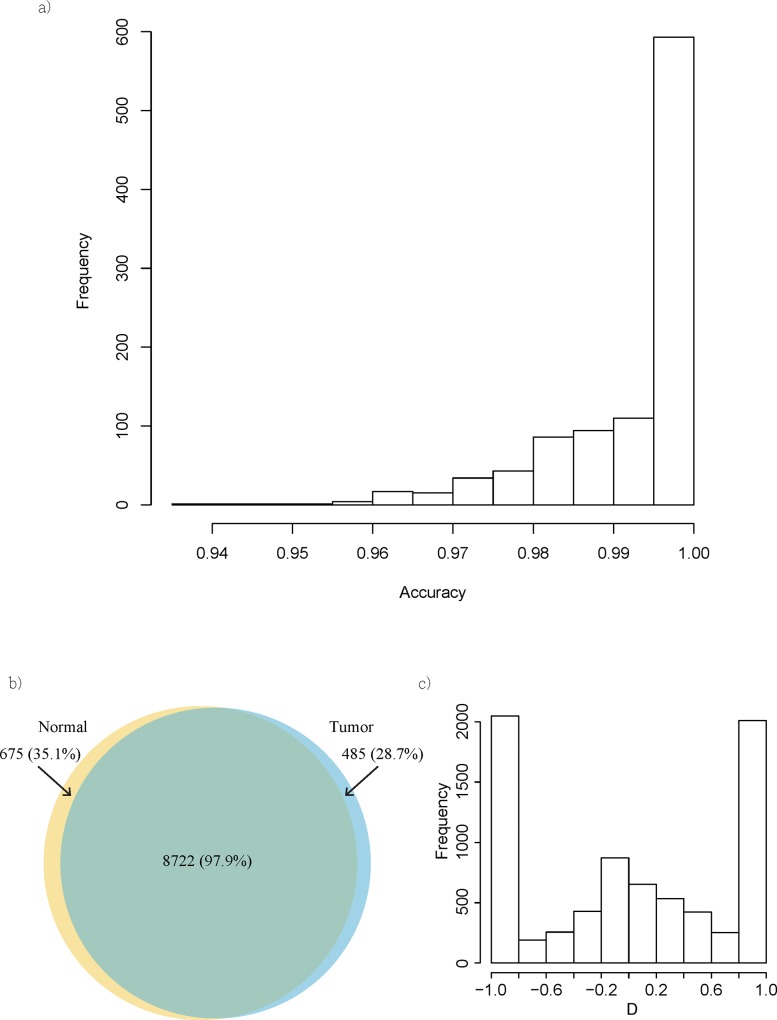
(a) Distribution the accuracy of NMD Classifier in 1,000 simulation experiments. (b) The numbers of NMDTs identified in the transcriptomes of paired normal-tumor tissues from lung adenocarcinoma; the numbers in the parentheses indicate the percentages of NMDTs that are annotated by Ensembl; (c) The distribution of relative expression level (D value) of NMDTs between tumor and normal tissue.

To evaluate the accuracy of NMD Classifier, we conducted a simulation study based on annotated human transcripts (Ensembl V75). We generated artificial transcript structure-altering events by randomly inserting or deleting an exon from a coding transcript, or changing the 5’ or 3’ boundary (or both) of an exon. Specifically, we randomly selected one coding transcript. Then a coding exon of this transcript was randomly selected and removed (random deletion). For random insertion, an intron between two coding exons was randomly selected from a transcript. Part of the intronic sequence was then “turned into” a coding exon. The length of this artificial exon followed the length distribution of real coding exons. A similar approach was applied to generate random boundary changes—an exon length and a target coding exon were randomly selected. If the random length was larger (or smaller) than that of the target exon, the target exon was extended (or abridged) at 5’, 3’, or both ends with equal probabilities. Only one transcript structure-altering event was generated in each transcript. Five thousand artificial transcripts were created in each simulation. NMD Classifier was then tested on the mock transcriptome. A total of one thousand simulations were conducted. Fig 3A shows that NMD Classifier could correctly identify an average of 99.3% of the NMD-causing transcript structural changes. Note that whether an artificial transcript represented an NMDT was determined by the 50-NT rule. Our results demonstrate that NMD Classifier was highly accurate in identifying NMD-causing transcript structural changes.

On potential concern in the above simulation study is that the “best partner” transcripts may have specific features not considered in the simulation. To address this issue, we compared the lengths and expression levels between the “best partner” transcripts and the other transcript isoforms from the same genes. Indeed, the “best partner” transcripts of NMDTs tended to be longer and less expressed (S1A and S1B Fig). However, the accuracies of NMD Classifier stayed at 98~99% regardless of length and expression level of the best partner transcript (S1C Fig).

Of note, here we only conducted simulations of single-exon transcript structural alterations (insertion/deletion or extension/shortening). In reality, multiple-exon alteration events might occur. However, the simulation of multiple-exon events is far more complex than single-exon simulations. Furthermore, in complex transcript structure-altering events, it is difficult to clarify the evolutionary/regulatory path leading to the emergence of an NMDT. Therefore, at this moment we may not be able to correctly assess the accuracy of NMD Classifier in detecting complex NMD-causing events. Nevertheless, complex transcript structural changes appear to be infrequent in NMDTs (Table 1), and thus may be less important.

**Table 1:** An exemplar classification of NMD events in paired normal-tumor tissues from a lung adenocarcinoma patient.

**Sample ID**	**ERR164502**	**ERR318893**
**Tissue Type**	Normal	Tumor
**NMD_ex**	2346	2328
**NMD_in**	3531	3452
**multi_NMD_ex**	414	399
**multi_NMD_in**	28	27
**A5SS**	1320	1341
**A3SS**	152	141
**A5SS+A3SS**	834	779
**NMD IR**	87	89
**nNMD IR**	40	38
**UTR_Diff**	573	543
**UTR_Diff_CDSdiff_NoFrameDiff**	39	34
**Complex**	33	36
**Total**	9397	9207

NMD_ex: exclusion of an exon; NMD_in: inclusion of an exon; multi_NMD_ex: exclusion of multiple exons; multi_NMD_in: inclusion of multiple exons; A5SS: the changes occurred at 5’ splicing site; A3SS: the changes occurred at 3’ splicing site; A5SS+A3SS: the changes occurred at both 5’ and 3’ (A3SS) splicing site; NMD_IR: intron retention that occurred in NMDT; nNMD_IR: intron retention that occurred in NMDT’s best partner; UTR_Diff: the NMDT and its best partner has identical coding sequences but different untranslated regions; UTR_Diff_CDSdiff_NoFrameDiff: the NMDT and its best partner has different coding sequences but no frame shift, the NMD may be caused by differences in untranslated regions; Complex: multiple types of transcript structure-altering events are involved.

### Application of NMD classifier to real data

We applied NMD Classifier to the transcriptomes of paired tumor-normal tissues from one lung adenocarcinoma patient. (Methods) [32]. Table 1 shows that each transcriptome included more than 9,000 NMDTs, with exon inclusion/exclusion-caused NMDTs (NMD_in and NMD_ex) representing the largest groups (~3,500 and ~2,300 NMDTs, respectively). The next largest group of NMDTs resulted from exon boundary changes (A3SS–alternative 3’splice site, A5SS–alternative 5’ splice site, and A3SS+A5SS), which represented ~2,300 NMDTs. Together NMD_in/ex and exon boundary changes accounted for ~87% of all of the NMDTs. The observation that NMD_in constituted the largest group was consistent with the result of a previous study [25]. About 6.6% (617 in 9,397) and 5.7% (525 in 9,207) of the NMDTs in normal and tumor tissue, respectively, were not annotated by Ensembl. This result indicated that most of the identified NMDTs in these transcriptomes have been previously annotated. However, caution should be taken because this might have resulted from insufficient RNA-sequencing depth [25] or the assembly approach adopted here (reference-based *de novo* assembly; see Methods). Meanwhile, the vast majority (92.8–94.7%) of the identified NMDTs were shared between tumor and normal tissue (Fig 3B). Nevertheless, hundreds of NMDTs were observed in tumor or normal tissue only, indicating that disease state-specific NMD events were present for lung adenocarcinoma. This observation is intriguing considering that the two transcriptomes were derived from the same organ of the same individual. Of note, even if tumor and normal tissue shared the same NMDTs, these NMDTs might have different expression levels. To illustrate this phenomenon, we defined a “D value” to measure the relative expression level of an NMDT between tumor and normal tissue (see Methods). D falls between -1 and +1, which indicate an NMDT is expressed exclusively in normal and tumor tissue, respectively. Fig 3C shows that ~2,000 NMDTs had their D values fall between -0.8 and -1.0, and another ~2,000 with D values between +0.8 and +1.0. Of note, a D value close to +1 indicated that the expression of the interested NMDT was close to zero in normal tissue, but its expression in the paired tumor tissue might not be high because D was an index of “relative expression”. Note that the large numbers of NMDTs at both D-value extremes might have resulted from very low expression levels of the relevant NMDTs. We thus screened out NMDTs with < 0.5 FPKM expression level in both of the samples. Indeed, the numbers of NMDTs at both extremes decreased (S2 Fig). The biomedical implications of these NMD events are worth further explorations.

## Discussion

In this study, we develop a convenient tool for identification and classification of NMD-causing transcript structural changes. Of note, these transcript structural alterations result from changes in RNA splicing pattern (exon inclusion/exclusion, exon boundary changes, intron retention…etc). What NMD Classifier does not address is mutation-caused generation of stop codon, which may also be a PTC in an NMDT. The detection of mutation-caused PTC requires another analysis flow, and is not included in NMD Classifier. However, if a mutation leads to a change in splicing pattern (such as a mutation at a splice site), NMD Classifier could detect this change in *de novo* assembled transcripts given an adequate number of exon junction reads.

We demonstrated by a simulation study that NMD Classifier could identity nearly 100% of the structural changes that led to NMD events. The small number of events not detected by NMD Classifier resulted from “erroneous selection” of best partner. Recall that NMD Classifier relies on pair-wise comparison between an NMDT and a best-matching coding transcript isoform from the same gene. We found that occasionally an annotated “coding transcript” selected as a best partner could be an NMDT according to the 50-NT rule. This inconsistency between 50-NT rule and annotation undermined the accuracy of NMD Classifier. Fortunately the number of such events was fairly small (<1% of all the analyzed cases). This observation suggests that selection of best partner is crucial for the accuracy of NMD Classifier. In case of *de novo* assembly, some of the assembled transcript structures may be less reliable. The qualities of transcript assembly can be examined by using RSEM-EVAL [33]. For the two transcriptomes examined in this study (ERR164502 and ERR318893), the *de novo* Cufflinks-assembled NMDTs actually had higher RSEM-EVAL scores than Ensembl-annotated NMDTs (S3 Fig). However, users are encouraged to examine the assembly qualities of their transcriptomes before applying NMD Classifier.

NMD Classifier can be applied to evolutionary and regulatory studies. For example, NMD Classifier can be used to identify NMD-causing events in one interested species. The evolutionary trajectory of these events can then be studied by using comparative approaches [25]. For regulatory and disease-oriented studies, one could compare the patterns and activities of NMD in different conditions such as diseased vs. normal tissues or the same tissue at different developmental stages. Such comparisons may lead to discoveries of the roles that NMD plays in important biological functions.

## Methods

### Preprocessing of transcriptome data

NMD Classifier takes a GTF or GFF annotation file as input to identify and classify NMDTs. These annotation files can be downloaded from Ensmebl (GTF) or NCBI (GFF). The user can also generate his/her own annotation file in GTF format from RNA-sequencing raw data. Firstly, the RNA-sequencing data (in FASTA or FASTQ format) should be mapped to the corresponding genome by using a sequencing read-mapping tool (e.g. TopHat or STAR) [34,35]. The mapping output file (in BAM or SAM format) then can be submitted to an assembly tool (e.g. Cufflinks or Trinity) [32,36] to yield a GTF file. A GTF file contains transcript structure information and genomic coordinates, which can be analyzed directed by NMD Classifier. In the current analysis, two transcriptomes (ERR164502 and ERR318893) were mapped to the human genome by using STAR [34](version 2.4.2) with default parameters. The mapping results were input to Cufflinks for reference-based *de novo* assembly and estimation of expression level. The example files are included in the downloadable NMD Classifier package.

### Analysis procedure

If the user uses standard annotation files downloaded from Ensembl or NCBI, NMDT annotations have been included in these files. However, if the user performs *de novo* assembly of transcripts by using tools such as Cufflinks, whether a transcript contains a PTC is unknown. In this case, NMD Classifier must predict the translation start site and the first in-frame stop codon. Since the transcriptional orientation was given by the assembler, NMD Classifier determined the coding region of a *de novo* assembled transcript by using three-frame conceptual translation. The reading frame that yielded the longest coding region was considered as the “correct” frame, and this longest coding region was defined as the main coding sequence of the *de novo* assembled transcript. The locations of the translation termination site and the last exon-exon junction could then be determined, and the distance in-between could be evaluated according to the 50-NT rule.

NMD Classifier classifies NMD-causing events based on the minimal evolution/regulation hypothesis. Particularly, it is hypothesized that an NMDT resulted from transcript structural alteration(s) of a coding transcript isoform, and that a minimal alteration was more likely to occur than a major one. Except in complex NMD events, the first structure-altering event that resulted in the non-rescued frameshift was considered as the cause of NMD. Therefore, for each NMDT, we selected the most similar coding transcript isoform (best partner) for comparison. Specifically, the coding transcript isoforms that shared translational start site with the interested NMDT were identified. Among these coding transcripts, the one that shared the largest proportion of nucleotides with the NMDT was chosen as the best partner of the interested NMDT.

**Fig 4:**
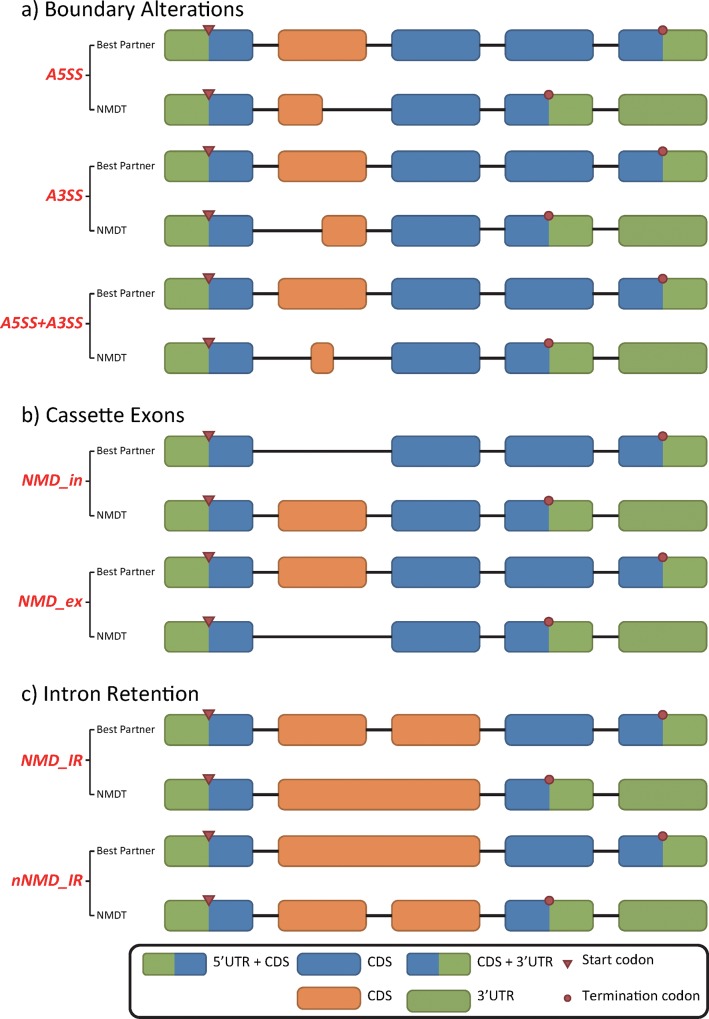
Alternative splicing events that result in NMDTs. (a) Changes at exon boundaries; (b) inclusion or exclusion of one or more coding exons; (c) Intron retention. Changes in untranslated regions and complex NMD events are not shown here. The orange boxes indicate the exon groups identified to be the cause of NMD events. NMDT: NMD transcript; nNMDT: non-NMD transcript; A5SS/A3SS: alternative 5’/3’ splice site; A5SS-A3SS: exon boundary changes at both 5’ and 3’ ends; NMD_in/ NMD_ex: inclusion/exclusion of an exon causes the NMD event; multi_NMD_in/multi_NMD_ex) inclusion/exclusion of multiple exons causes the NMD event; NMD_IR/ nNMD_IR: intron retention in the NMDT/non-NMDT causes the NMD event; CDS: coding sequence; UTR: untranslated region.

Based on pair-wise comparisons between NMDTs and their best partners, NMD-causing events were classified into five groups: (i) boundary alterations: the changes occurred at either 5’ (A5SS) or 3’ (A3SS) splicing site, or both (A5SS-A3SS) (Fig 4A); (ii) cassette exons: inclusion (NMD_in) or exclusion (NMD_ex) of an exon or multiple exons (multi_NMD_in, multi_NMD_ex) resulted in NMD (Fig 4B); (iii) intron retention: intron retention that occurred in NMDT (NMD_IR) or in its best partner (nNMD_IR) (Fig 4C); (iv) UTR alterations: the NMDT and its best partner had identical coding sequences but different UTRs; and (v) complex events: the difference between an NMDT and its best partner included multiple types of transcript structural changes. An exemplar complex event comprises an exon inclusion in the NMDT and an intron retention event in the best partner.

**Fig 5:**
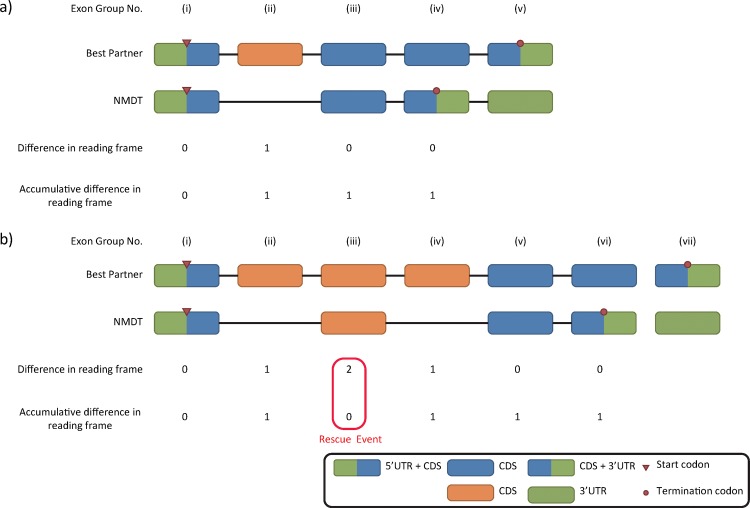
Examples of how NMD Classifier identifies NMD-causing event by calculating differences in reading frame between an NMDT and its best partner. (a) Insertion of a single exon [exon group (ii)] causes a one-base frameshift and therefore an NMD event; (b) A one-base frameshift occurs at exon group (ii) but is offset downstream at group (iii). NMD Classifier thus continues to scan for the next frameshift event, which occurs at group (iv). This latter frameshift is maintained throughout to the stop codon, and is identified as the NMD-causing event.

Specifically, for each NMDT and its best partner, the genomic coordinates of each exon (retrieved from the GTF file) were compared. Exons whose corresponding genomic regions overlapped with each other by at least one nucleotide were grouped together as an “exon group”. For example, if an exon of an NMDT was located at genomic coordinates 100–500, whereas an exon of its best partner was located at 300–700, these two exons were considered as an exon group that spanned coordinates 100–700. In another example, if an exon of an NMDT was located at 2000–2500, and two exons of its best partner were located at 1800–2200 and 2300–2700, respectively. The resulting exon group would span coordinates 1800–2700. In each exon group upstream of the PTC, the difference in reading frame between NMDT and its best partner was calculated and summed up from 5’ to 3’. Except in complex events, the first (most upstream) frameshift event was considered as an NMD-causing event (Fig 5A) unless the frameshift was “rescued” by a second, downstream event. If a “rescue” event occurred, the first frameshift event downstream of the “rescue” event was regarded as the NMD-causing event (Fig 5B).

### Test transcriptome data source and data processing

Two transcriptomes (ERR164502 and ERR318893) from lung adenocarcinoma were downloaded from the Gene Expression Omnibus (GEO) database [37] with accession number GSE40419. The transcriptomes were mapped to the human genome (Hg19, Ensembl V75) by using STAR [34]. The transcripts were assembled with the reference-based *de novo* assembly function of Cufflinks [32], and then analyzed by using NMD Classifier.

To analyze the relative expression level of NMDTs in tumor and normal tissues, we defined the “D” value as follows:

$$D=\left\{ \begin{array}{l} 0\;\mathrm{if the FPKM values in normal and tumor tissue are both}\;0;\\ \frac{E_{T}-E_{N}}{E_{T}+E_{N}}\;\mathrm{otherwise} \end{array}\right.$$ (1)

Where *E*_*T*_ and *E*_*N*_, respectively, indicated the expression level (in FPKM) of the interested NMDT in tumor and normal tissue.

## Supporting information

S1 Fig Distributions of relative transcript length (left panel) and relative expression level (right panel) of best partners and the other transcripts in ERR164502 (a) and ERR318893 (b). The accuracies of NMD Classifier in the simulation study (c) across different relative transcript lengths (left panel) and relative expression levels (right panel).
(TIFF)

S2 Fig Distribution of D values for transcripts with ≧ 0.5 FPKM expression level in at least one sample. (TIFF)

S3 Fig Distributions of RSEM-EVAL score (a) and expression level (b) of Ensembl-annotated NMDTs and *de novo* assembled NMDTs in two test samples (left panel: ERR164502; right panel: ERR318893).
(TIFF)

S1 Table Transcript support levels of NMDTs (XLSX)
