# Supplementary figures and images for: NMD Classifier: A reliable and systematic classification tool for nonsense-mediated decay events
Source: PLoS One. 2017 Apr 3;12(4):e0174798. doi: 10.1371/journal.pone.0174798 (PMC5378362; doi:10.1371/journal.pone.0174798)

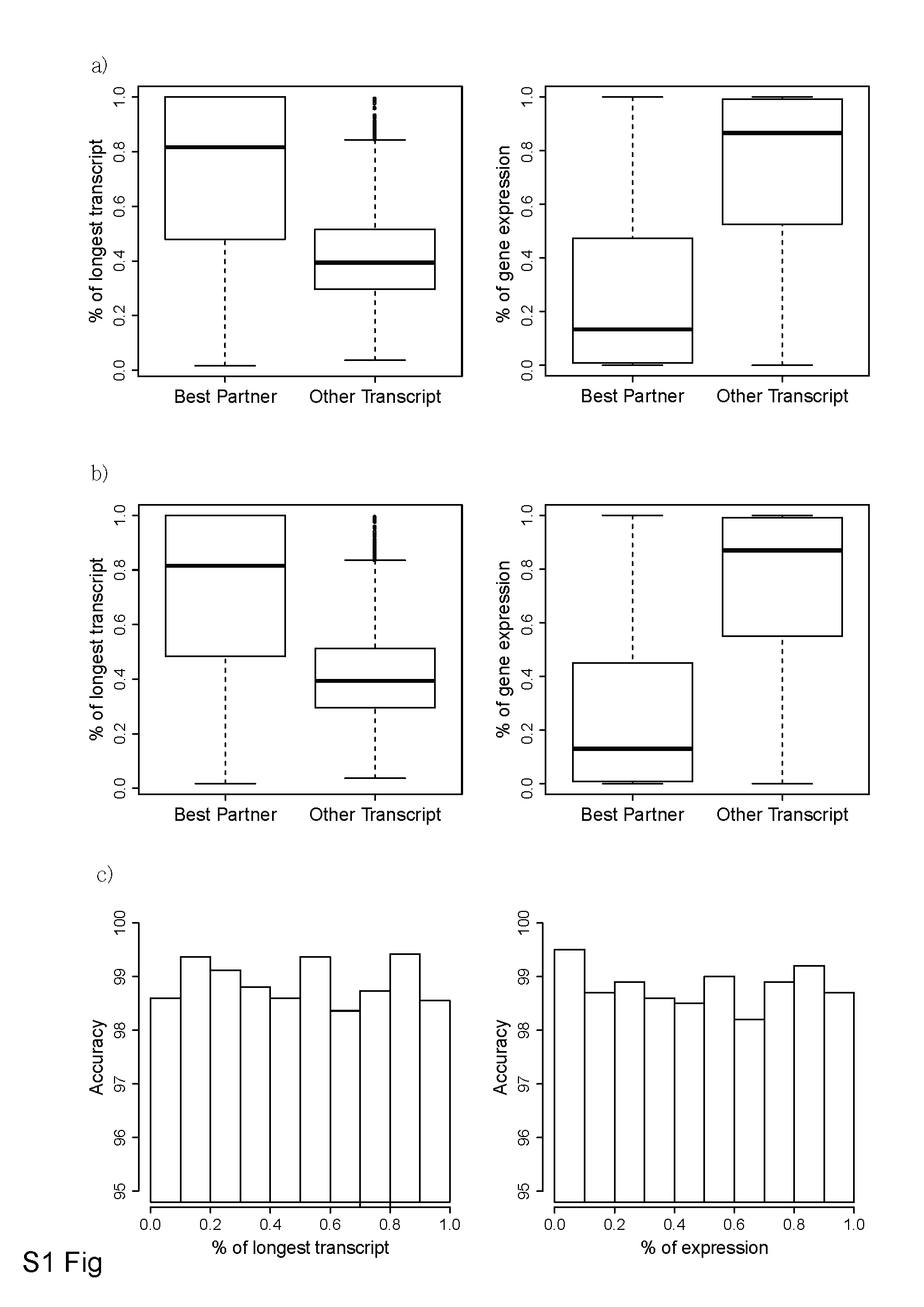

Supplement: S1 Fig — Distributions of relative transcript length (left panel) and relative expression level (right panel) of best partners and the other transcripts in ERR164502 (a) and ERR318893 (b). The accuracies of NMD Classifier in the simulation study (c) across different relative transcript lengths (left panel) and relative expression levels (right panel). (TIFF) [file pone.0174798.s001.tiff]

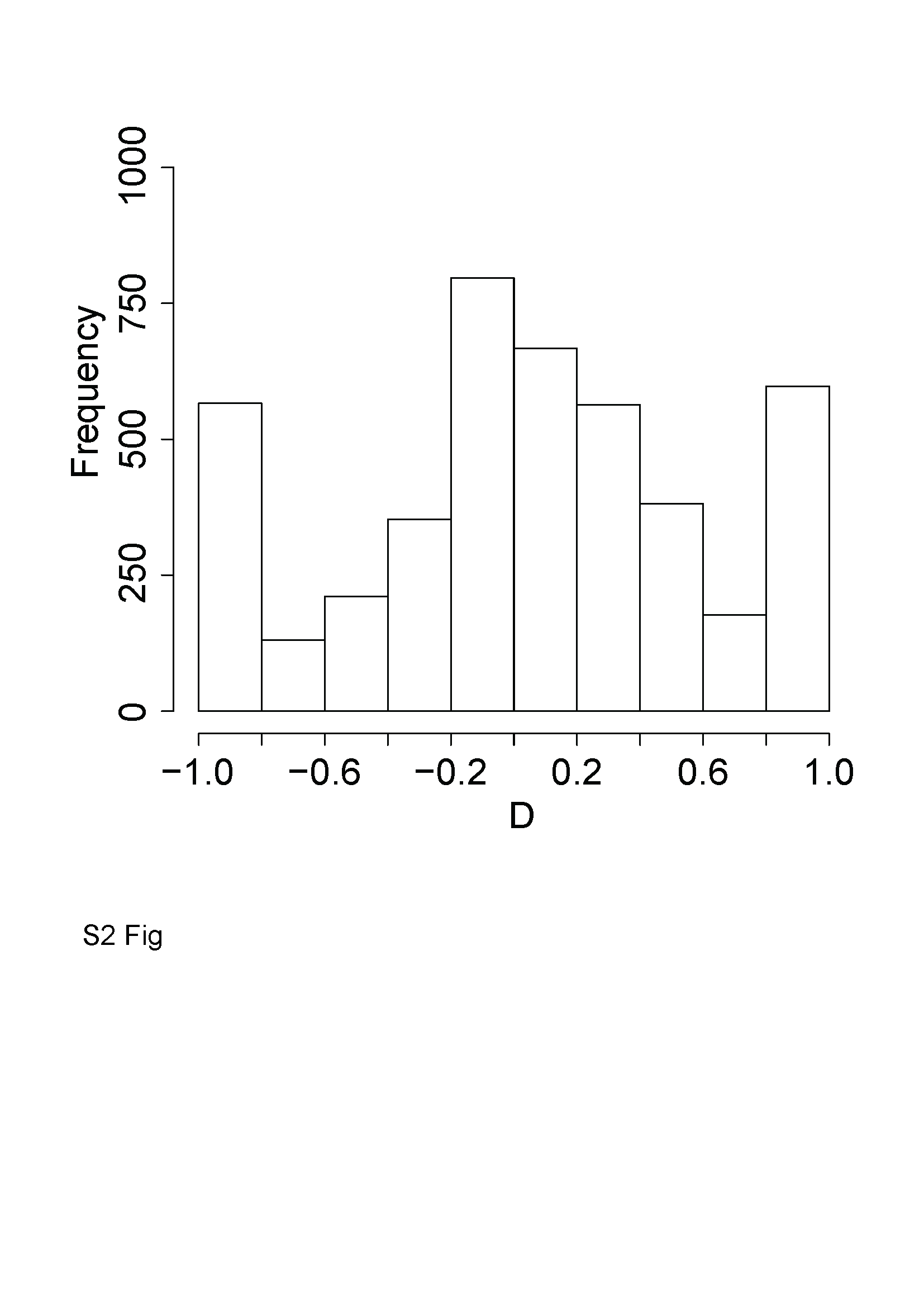

Supplement: S2 Fig — (TIFF) [file pone.0174798.s002.tiff]

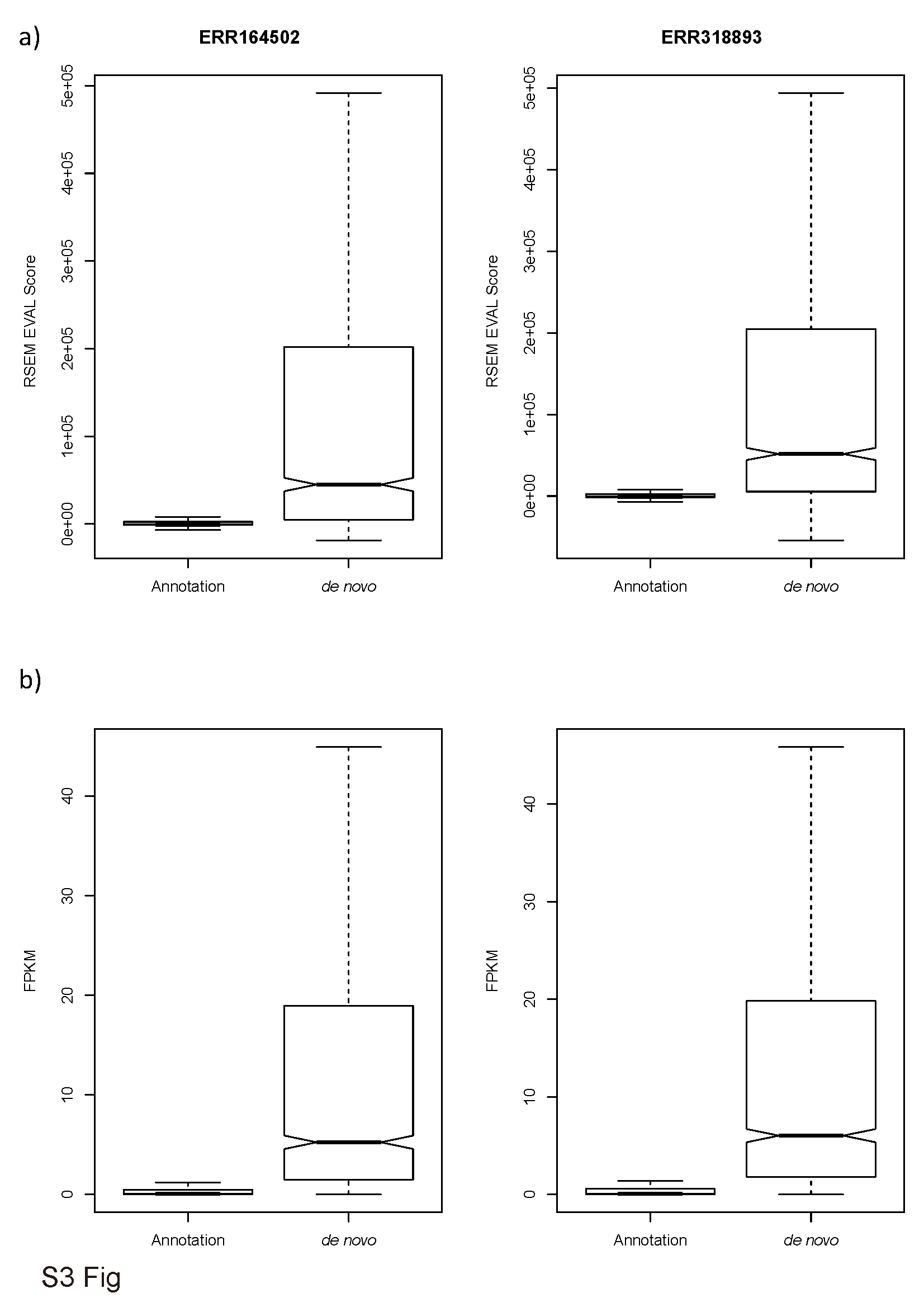

Supplement: S3 Fig — Distributions of RSEM-EVAL score (a) and expression level (b) of Ensembl-annotated NMDTs and de novo assembled NMDTs in two test samples (left panel: ERR164502; right panel: ERR318893). (TIFF) [file pone.0174798.s003.tiff]
